# A dual role of the extracellular domain of *Drosophila* Crumbs for morphogenesis of the embryonic neuroectoderm

**DOI:** 10.1242/bio.031435

**Published:** 2018-01-15

**Authors:** Shradha Das, Elisabeth Knust

**Affiliations:** Max-Planck-Institute of Molecular Cell Biology and Genetics, Pfotenhauerstrasse 108, 01307 Dresden, Germany

**Keywords:** Notch, Actomyosin, Polarity, Adhesion, Neurogenesis

## Abstract

Epithelia are highly polarised tissues and several highly conserved polarity protein complexes serve to establish and maintain polarity. The transmembrane protein Crumbs (Crb), the central component of the Crb protein complex, is required, among others, for the maintenance of polarity in most epithelia in the *Drosophila* embryo. However, different epithelia exhibit different phenotypic severity upon loss of *crb*. Using a transgenomic approach allowed us to more accurately define the role of *crb* in different epithelia. In particular, we provide evidence that the loss of epithelial tissue integrity in the ventral epidermis of *crb* mutant embryos is due to impaired actomyosin activity and an excess number of neuroblasts. We demonstrate that the intracellular domain of Crb could only partially rescue this phenotype, while it is able to completely restore tissue integrity in other epithelia. Based on these results we suggest a dual role of the extracellular domain of Crb in the ventral neuroectoderm. First, it is required for apical enrichment of the Crb protein, which in turn regulates actomyosin activity and thereby ensures tissue integrity; and second, the extracellular domain of Crb stabilises the Notch receptor and thereby ensures proper Notch signalling and specification of the correct number of neuroblasts.

## Abstract (precis)

**Summary:** Using a transgenomic approach we determine specific roles of the intra- and extracellular domain of the Crumbs protein for the maintenance of apico-basal epithelial polarity and epithelial morphogenesis in *Drosophila* embryos.

## INTRODUCTION

Epithelia are highly polarised tissues that can be specialised for protection, absorption, secretion, transport or sensory perceptions. Hence, mechanisms controlling polarity and integrity of epithelial tissues are important for shaping tissues during development. In addition, maintaining polarity is essential for tissue homeostasis in adult organisms, which is reflected by the fact that 80-90% of all cancer types derive from epithelia (Cao et al., 2015; Grifoni et al., 2013; Laprise, 2011; McCaffrey and Macara, 2011; Tellkamp et al., 2014). Therefore, unravelling the basis of epithelial polarity and the mechanisms required to maintain tissue integrity is crucial to understand the origin of various diseases. *Drosophila* embryonic epithelia are excellent model tissues to study the genetic, molecular and cellular basis of development and maintenance of polarity. In particular, studies focussing on the embryonic epidermis have provided deep insight into the regulation of tissue polarity and integrity. The epidermis is subject to mechanical stress during various morphogenetic events, such as germ band extension or retraction, yet it is maintained as a properly polarised, coherent mono-layered sheet during these processes. Work from many groups have shown that elaborated adherens junctions (AJs), in particular the zonula adherens (ZA), a belt-like structure encircling the apex of the cell, is instrumental to provide adhesive strength in order to counteract mechanical forces, but at the same time is flexible to allow tissue movements and changes during morphogenesis (Harris and Tepass, 2010; Macara et al., 2014; Oda and Takeichi, 2011). Formation and maintenance of the ZA depends, among others, on proper apico-basal cell polarity. Polarity is established and maintained by a crosstalk between the polarised trafficking machinery and a polarised cytoskeleton, orchestrated by a sophisticated interplay of proteins forming the ‘epithelial polarity program’ (Rodriguez-Boulan and Macara, 2014). Three major evolutionarily conserved protein modules, the apical Par- and Crumbs (Crb)-complexes and the baso-lateral Lgl/Scrib/Dlg-module, act as key regulators of epithelial polarity and tissue integrity in various epithelia (reviewed in Campanale et al., 2017; Flores-Benitez and Knust, 2016; Macara et al., 2014; Tepass, 2012).

Initially discovered in a screen for genes with sequence homology to the neurogenic genes *Notch* and *Delta* (Knust et al., 1987), *Drosophila* Crb, the founding member of the Crb-complex, subsequently emerged as an evolutionarily conserved polarity regulator conserved from worms to human. *crb* genes encode type 1 transmembrane proteins, which are enriched at the sub-apical region right apical to the ZA. The cytoplasmic domains of Crb proteins are highly conserved and characterised by a C-terminal PDZ- (PSD-95, Dlg, ZO-1)-binding motif (PBM) and an N-terminal FERM- (4.1, ezrin, radixin, moesin)-domain binding motif (FBM). Similarly, the binding partners are highly conserved, including the PDZ-proteins Stardust (Sdt) and *Dm*Par6 of *Drosophila* and their mammalian orthologues MPP5/PALS1 and Par6, respectively, and the FERM-proteins Moesin and Yurt/Mosaic eyes-like 1 (YMO1/EPB41L5) (Gosens et al., 2007; Le Bivic, 2013; Tepass, 2009). Loss or increased levels of Crb lead to disruption of apico-basal polarity and a breakdown of the mono-layered embryonic epithelial structure, followed by embryonic lethality (Tepass et al., 1990; Wodarz et al., 1993, 1995). This suggests that Crb levels at the apical membrane are crucial for the maintenance of polarity and tissue integrity. Multiple mechanisms contribute to maintain appropriate levels of Crb at the subapical region, including trafficking to and from the apical plasma membrane (Blankenship et al., 2007; Lin et al., 2015; Lu and Bilder, 2005; Pocha and Wassmer, 2011; Shivas et al., 2010; Zhou et al., 2011), as well as stabilisation at the membrane via the intra- or extracellular domain (Bachmann et al., 2001; Hong et al., 2001; Kempkens et al., 2006; Letizia et al., 2013).

Whereas *Drosophila* contains only one *crb* gene, *Caenorhabditis elegans*, zebrafish, mouse and human genomes encode more than one *crb* orthologues. In all *crb* genes described so far, the short intracellular domain (ICD) is highly conserved. In contrast, based on the extracellular domain (ECD), *C. elegans* and vertebrate *crb* genes can be subdivided into two groups: one group (*Crb1* and *Crb2*) encodes proteins with a large ECD similar as the one found in *Drosophila* Crb, which is characterised by an array of variable numbers of epidermal growth factor (EGF)-like repeats interspersed by repeats with similarity to the globular domain of Laminin A. The second group (*Crb3*) encodes transmembrane proteins containing a very short ECD with no similarity to that of the first group. While many Crb-dependent functions, such as regulation of polarity and cytoskeleton activity, could be allocated to the short ICD, the role of the long ECD is still elusive. Earlier studies have suggested that homophilic interactions between the ECDs are responsible for stabilising the protein at the membrane, thus ensuring proper protein levels and maintenance of cell polarity in the embryonic epidermis (Letizia et al., 2013; Thompson et al., 2013). In addition, anisotropic distribution of Crb protein in neighbouring cells, mediated by the ECDs, has been proposed to be required for the recruitment of a circumferential actomyosin cable in cells with low Crb, which drives tissue invagination during tube formation in the embryo (Röper, 2012). In the zebrafish eye, homo- and heterophilic interactions between the ECDs of Crb2a/Crb2b in cone photoreceptor cells are required for proper patterning of the retina (Raymond et al., 2014; Zou et al., 2012; reviewed in Pocha and Knust, 2013; Thompson et al., 2013). In *Drosophila* eye and wing imaginal discs the ECD has been implicated in growth control (Richardson and Pichaud, 2010). Recently, we could show that the ECD of Crb stabilises the Notch-receptor in the apical membrane, thus preventing ligand-independent Notch signalling during vein formation in the pupal wing (Nemetschke and Knust, 2016).

Interestingly, although *crb* is expressed in all embryonic epithelia derived from the ectoderm, defects in epithelia of *crb* mutant embryos range from complete disintegration and widespread apoptosis (e.g. in some parts of the epidermis) to no obvious polarity defect at all (e.g. in the hindgut) (Tepass and Knust, 1990). The defects can even differ in the same tissue. For example, the ventral epidermis was shown to be more affected than the dorsal epidermis upon loss of *crb* (Kolahgar et al., 2011). The analysis to explain the different phenotypic severity has been hampered by two facts. (i) Most results obtained so far were based on overexpression studies of full-length Crb proteins or just part of it, using the Gal4/UAS system. Thereby it was shown that the membrane-bound ICD is able to restore polarity and tissue integrity in many epithelia to the same degree as the full-length protein. However, in this experimental set-up, neither the ICD nor the full-length protein can rescue embryonic lethality of *crb* mutant embryos (Klebes and Knust, 2000; Wodarz et al., 1995). In addition, expression of rescue constructs using the Gal4/UAS system results in excessive protein, thus hindering the analysis of tissue sensitivity towards differential levels of Crb. In contrast, a fosmid encoding the complete genomic locus of *crb* (called *foscrb*), the expression of which is under endogenous control, rescues all aspects associated with loss of *crb* and gives rise to viable and fertile adults (Klose et al., 2013). (ii) The second impediment to achieve an in-depth understanding of epithelia-specific roles of Crb is the lack of appropriate hypomophic alleles. Embryos homozygous mutant for amorphic *crb* alleles display severe defects in early embryogenesis leading to massive apoptosis, which makes it difficult to unveil tissue-specific functions of Crb.

In order to understand the full potential of the ICD and ECD of Crb and their requirements in different epithelia, we engineered *foscrb* to create flies containing either *foscrb_ICD_* or *foscrb_ECD_*, which encode the membrane-bound ICD and ECD, respectively. We show that fosmid-based expression of the ICD not only rescues polarity defects in most epithelia of *crb* mutant embryos, but also, and in contrast to results obtained from overexpression studies, is sufficient for proper invagination and morphogenesis of epithelial tubes and, strikingly, for viability in about 50% of cases. In addition, we provide data to show that the strong phenotype of the ventral epidermis of *crb* mutant embryos can be traced back to a neurogenic phenotype due to the development of an excess of neuroblasts. This phenotype could only partially be rescued by expressing the ICD only. This suggests an essential role of the ECD for maintaining the integrity of the neuroectoderm. Here, we propose two mechanisms by which the ECD mediates this function. First, it is required for apical enrichment of Crb, which, in turn, controls actomyosin activity; and second, the ECD ensures apical Notch localization and proper signalling in the neuroectoderm, and thus prevents the formation of supernumerary neuroblasts.

## RESULTS

### The extracellular domain of Crb is not essential for embryogenesis

**Fig. 1.**
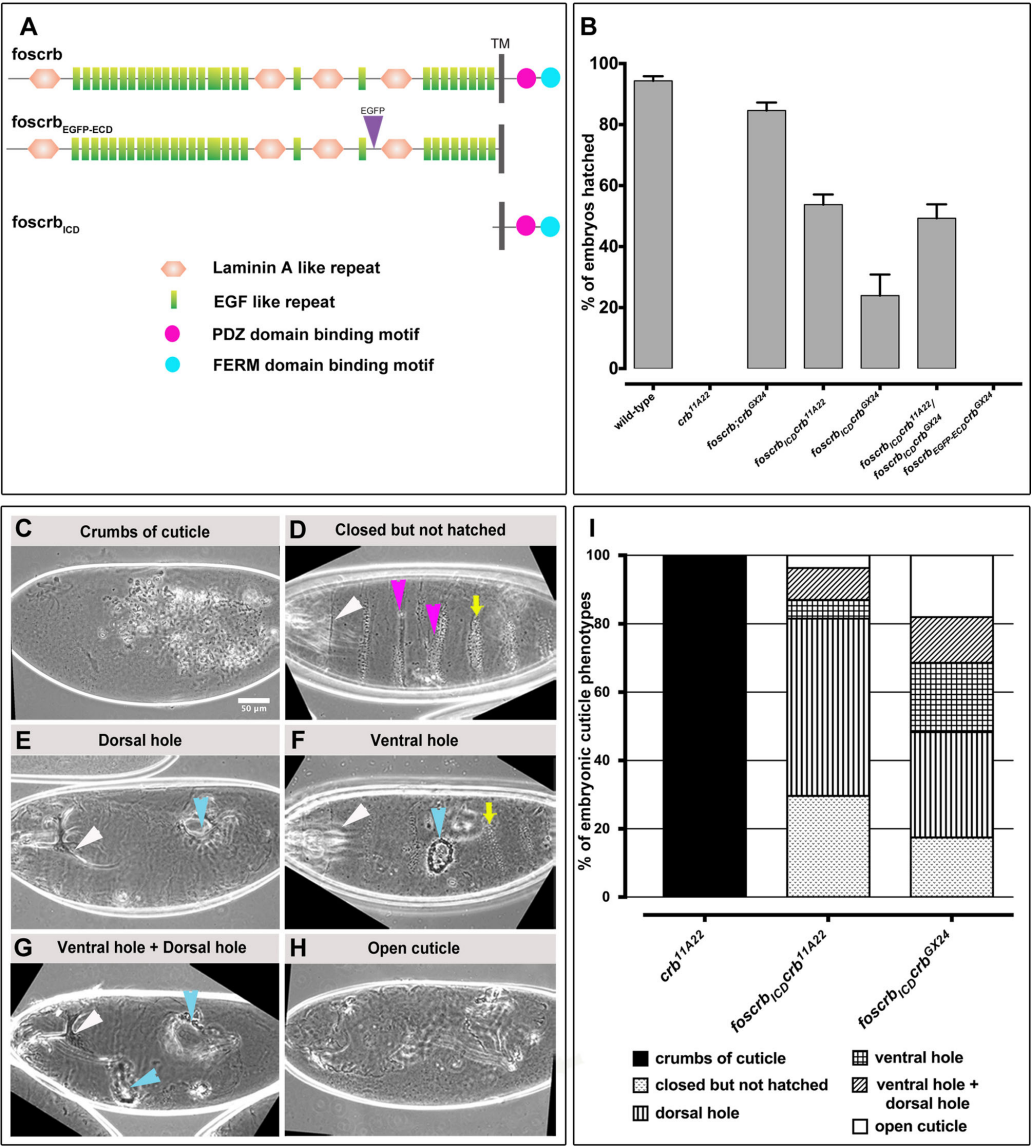
**The ICD of Crb restores epithelial integrity of *crb* mutant embryos.** (A) Schematic representation of the fosmids. Depicted proteins are based on the Crb-PA isoform (2.146 amino acids). Adopted from Klose et al. (2013). (B) Analysis of embryonic lethality. The graph represents the percent of embryos that hatch. *N*>1000 embryos. The experiment was repeated 5 times. Error bars show standard error of the mean. (C-I) Classification of cuticle phenotypes of unhatched embryos. White arrowhead (D,E,F,G), intact head structure; yellow arrow (D,F), intact denticle belts; magenta arrowheads (D), fused or lost denticle belts; cyan arrowhead (E,F), dorsal/ventral hole, respectively; cyan arrowheads (G), ventral and dorsal hole. Scale bar: 50 μm. (J) Quantification of cuticle phenotypes. *N*>300 embryos. The experiment was repeated 3 times.

Gal4-mediated overexpression of the membrane-bound intracellular domain of Crb can suppress the phenotype of *crb* mutant embryos to the same degree as overexpression of a full-length Crb protein (Klebes and Knust, 2000; Wodarz et al., 1995). In contrast, fosmids containing the entire *crb* locus (*foscrb* and *foscrb-EGFP*) can completely rescue the lethality of amorphic *crb^11A22^* and *crb^GX24^* alleles (Klose et al., 2013). In order to dissect the functions of the intracellular and extracellular domain of Crb (here called ICD and ECD, respectively) in embryonic development under physiological conditions, we modified *foscrb* and *foscrb_EGFP_* to generate two transgenes. *foscrb_ICD_* encodes a membrane bound ICD of Crb, in which the ECD was deleted with the exception of the C-terminal 8 amino acids (KEAYFNGS). *foscrb_ECD-EGFP_* encodes an Enhanced Green Fluorescent Protein (EGFP)-tagged membrane-bound ECD, in which most of the ICD has been deleted with the exception of the N-terminal 7 amino acids (MARNKRAT) (Fig. 1A). *foscrb_ICD_*, *foscrb_ECD-EGFP_* as well as a fosmid encoding EGFP-tagged full-length Crb proteins (*foscrb_EGFP_*) were integrated into the VK00033 landing site (chromosomal location 65B2 on 3L). The three transgenic lines were recombined with *crb^11A22^* and *crb^GX24^* alleles. *foscrb_EGFP_ crb^11A22^* and *foscrb_EGFP_ crb^GX24^* were viable and fertile and could be maintained as homozygous stocks, similar as *foscrb;crb^GX24^* (Klose et al., 2013).

To test the rescuing activity of the ICD and ECD of Crb, we analysed the percentage of homozygous *crb* mutant larvae carrying two copies of the respective transgenes (Fig. 1B). While none of the homozygous *crb^11A22^* embryos hatched, 95% of the wild-type embryos and 85% of *crb^GX24^* mutant embryos expressing *foscrb* hatched. *foscrb_ECD-EGFP_* completely failed to rescue homozygous *crb* null embryos (Fig. 1B). Strikingly, 50% and 25% of homozygous *crb^11A22^* and *crb^GX24^* embryos, respectively, that express *foscrb_ICD_*, hatched, but these larvae died as first or second instar. Since 50% of *crb^11A22^*/*crb^GX24^* transheterozygous embryos expressing *foscrb_ICD_* hatched, the lower hatching rate in *foscrb_ICD_ crb^GX24^* embryos is likely due to the genetic background. Therefore, further analyses were carried out with *foscrb_ICD_ crb^11A22^* embryos (referred to as *foscrb_ICD_ crb* henceforth).

The *crb* locus was named according to its cuticle phenotype, which reveals only ‘crumbs’ of cuticle instead of a continuous cuticle, due to a complete breakdown and death of the epidermis (Jürgens et al., 1984; Tepass and Knust, 1990) (Fig. 1C). To further determine to what extent the different transgenes could suppress the *crb* mutant phenotype in those embryos that did not hatch, we quantified the cuticle phenotypes of embryos with different genotypes (Fig. 1C-H and I). Homozygous *foscrb_ICD_ crb^11A22^* and *foscrb_ICD_ crb^GX24^* embryos showed variable cuticle phenotypes. 20-30% of embryos formed a continuous, nearly wild-type cuticle (Fig. 1D) with intact head structures (white arrowhead) and denticle belts (yellow arrow), which were occasionally merged or absent (magenta arrowheads). Others showed a dorsal hole (cyan arrowhead in Fig. 1E), a ventral hole (cyan arrowhead in Fig. 1F) or ventral and dorsal holes (cyan arrowheads in Fig. 1G). *foscrb_ECD-EGFP_ crb^11A22^* embryos did not show any rescuing activity and resemble *crb* mutant embryos without any transgene (data not shown). Together these results show that the ICD of Crb is indispensable for the function of Crb in maintaining embryonic epithelial integrity, but is insufficient to ensure robustness in completion of successful embryogenesis and proper larval development.

### The ICD of Crb restores integrity of most embryonic epithelia

**Fig. 2.**
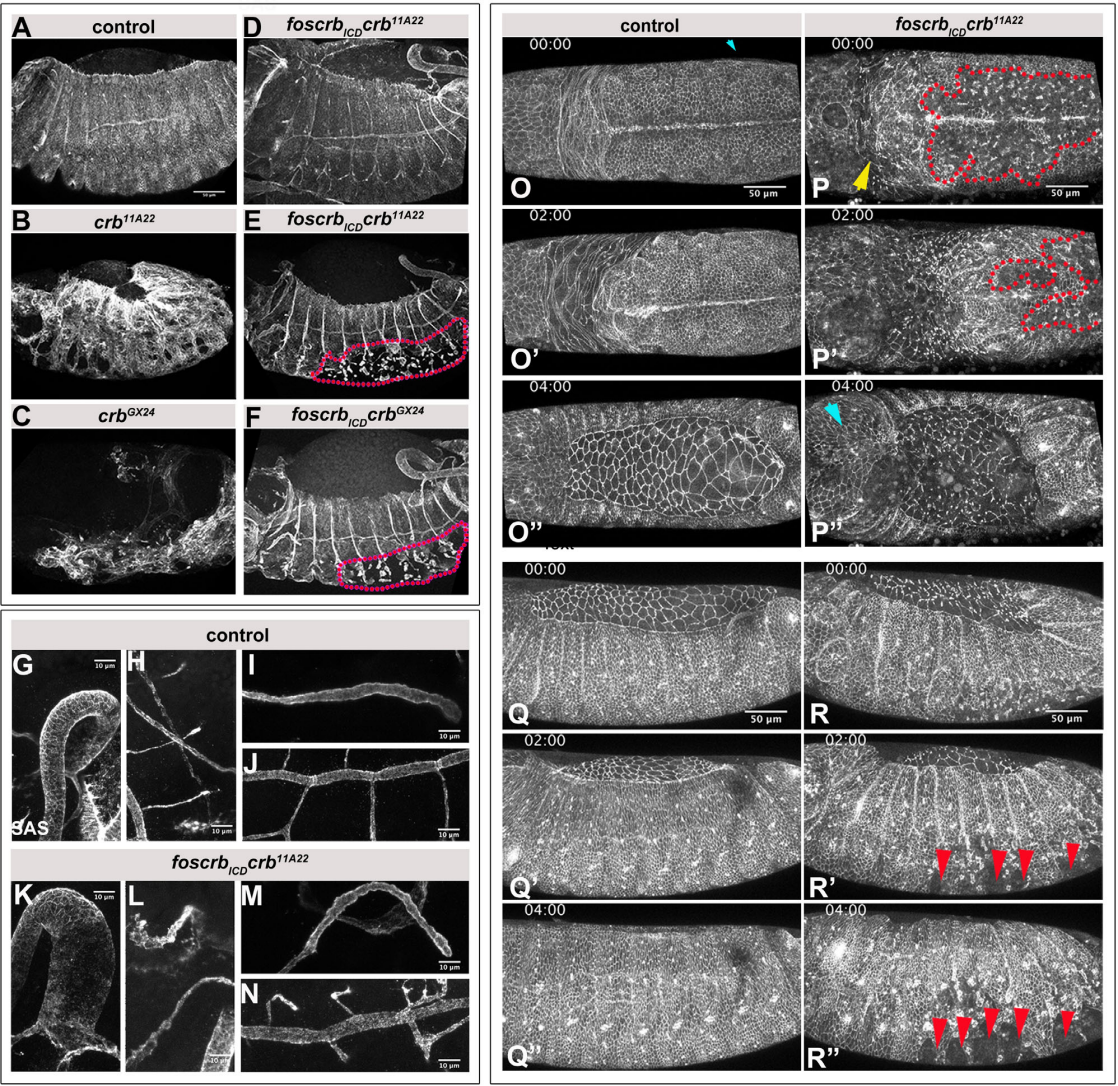
**The Crb ICD is sufficient for tissue integrity of most embryonic epithelia.** (A-F) Stage 12-13 embryos, stained for SAS. Dotted lines in E and F mark the disintegrated ventral epidermis. Dorsal is up, anterior left. Scale bar: 50 μm. The experiment was repeated 3 times. (G-N) Stage 12-13 *foscrb; crb^GX24^* control (G-J) and *foscrb_ICD_ crb^11A22^* (K-N) embryos, stained with anti-SAS. Polarity of epithelial tubes is restored in the hindgut (G,K), the Malpighian tubules (H,L), the salivary gland (I,M) and the trachea (J,N). Scale bar: 10 μm. (O-R″) Stills of time lapse movies of endogenously tagged *D*E-Cadherin-GFP lines. Dorsal (O-P″) and lateral (Q-R″) views of *foscrb; crb^GX24^* control and *foscrb_ICD_ crb^11A22^* embryos. Red dotted lines in P,P′ mark the disintegrated ventral epidermis. Yellow arrow in P, disintegrated head epidermis; cyan arrowhead in P″, recovered head epidermis; red arrowheads in R′,R″, ‘wounds’ in ventral epidermis. Anterior is to the left. Scale bar: 50 μm. The experiment was repeated 3 times.

The dorsal and ventral holes in the cuticle of *foscrb_ICD_ crb* mutant embryos implied that the integrity of some epithelia was not completely restored in the absence of ECD. To corroborate this assumption, epithelial integrity was analysed in stage 12/13 embryos using the apical marker Stranded-at-second, SAS (Fig. 2). This revealed that the sheet-like structure and integrity of the epidermis as seen in control embryos (Fig. 2A) is completely lost in *crb^11A22^* (Fig. 2B) and *crb^GX24^* (Fig. 2C) embryos. Strikingly, some *crb* embryos expressing *foscrb_ICD_* showed a completely restored epidermis (Fig. 1D). Other embryos had intact head and dorsal epidermis, but exhibited a disintegrated ventral epidermis (magenta region in Fig. 2E,F). In all cases, invagination and development of epithelial tubes, such as the hindgut, the Malpighian tubules, the salivary glands and the tracheae occurred properly in *crb* embryos expressing *foscrb_ICD_* (Fig. 2G-N).

To better understand the development of the mutant phenotype in the ventral epidermis of *foscrb_ICD_ crb* mutant embryos, we imaged endogenously tagged *D*E-Cadherin-GFP in embryos of the respective genotypes (Fig. 2O-O″,P-P″,Q-Q″,R-R″; Movies 1 and 2). The defects in the ventral epidermis (region demarcated by red dots in Fig. 2P,P′) were already evident in embryos prior to the onset of germ band retraction in *foscrb_ICD_ crb* mutant embryos. In addition, the head epidermis displayed loss of *D*E-Cadherin already at this stage (yellow arrow in Fig. 2P). The defects in the ventral epidermis (Fig. 2P′) persisted as germ band retraction proceeded, whereas the head epidermis seemed to recover by the end of germ band retraction (cyan arrow in Fig. 2P″). During the stage of dorsal closure, the ventral epidermis of *foscrb_ICD_ crb* mutant embryos displayed multiple, ‘wound’-like gaps (red arrows in Fig. 2R′ and R″), while the epidermis stayed intact during germ band retraction and dorsal closure of control embryos (Fig. 2O-O″ and Q-Q″). Closer examination of the ventral epidermis in the mutant embryos revealed that the cells gradually rearranged, resulting in the transformation of a mono-layered sheet into multiple, ‘cyst’-like structures (Fig. S1A-A″″, Movie 3). As development proceeds, the ventral epidermis of some of the *crb* mutant embryos expressing *foscrb_ICD_* ripped apart (Fig. S1D-D″). In other embryos of the same genotype, however, the epidermis sealed these gaps later during embryogenesis (Fig. S1C-C″, Movie 4).

We reasoned that the dorsal hole detected in cuticle preparations of *foscrb_ICD_ crb* mutant embryos may be due to defects in the development of the amnioserosa, an extraembryonic tissue that covers the dorsal side of the embryo. During dorsal closure, the lateral epidermis moves dorsal wards, while the amnioserosa is internalised. Finally, zippering of the two sides of the epidermis closes the embryo dorsally (Fig. S2A-A″″) (reviewed in Hayes and Solon, 2017). While in *crb* mutant embryos the amnioserosa disintegrates already during germband extension (Grawe et al., 1996; Tepass, 1996), expression of the ICD of Crb in these embryos prevents the collapse of the amnioserosa at early stages. However, at the end of germ band extension and later on, defects in *D*E-cadherin staining were obvious (Fig. S2B-B″). In addition, the zippering process is impaired in a subset of mutant embryos. Posterior zippering is not initiated (green arrows in Fig. S2B), and, as a consequence, dorsal closure fails (Fig. S2B-B″″).

These results demonstrate that the ICD of Crb is sufficient for maintaining integrity of the dorsal epidermis and for invagination of epithelial tubes, but is insufficient to maintain integrity of the ventral epidermis and the amnioserosa.

### Apico-basal polarity is restored in the epidermis of *crb* embryos expressing the intracellular domain

**Fig. 3.**
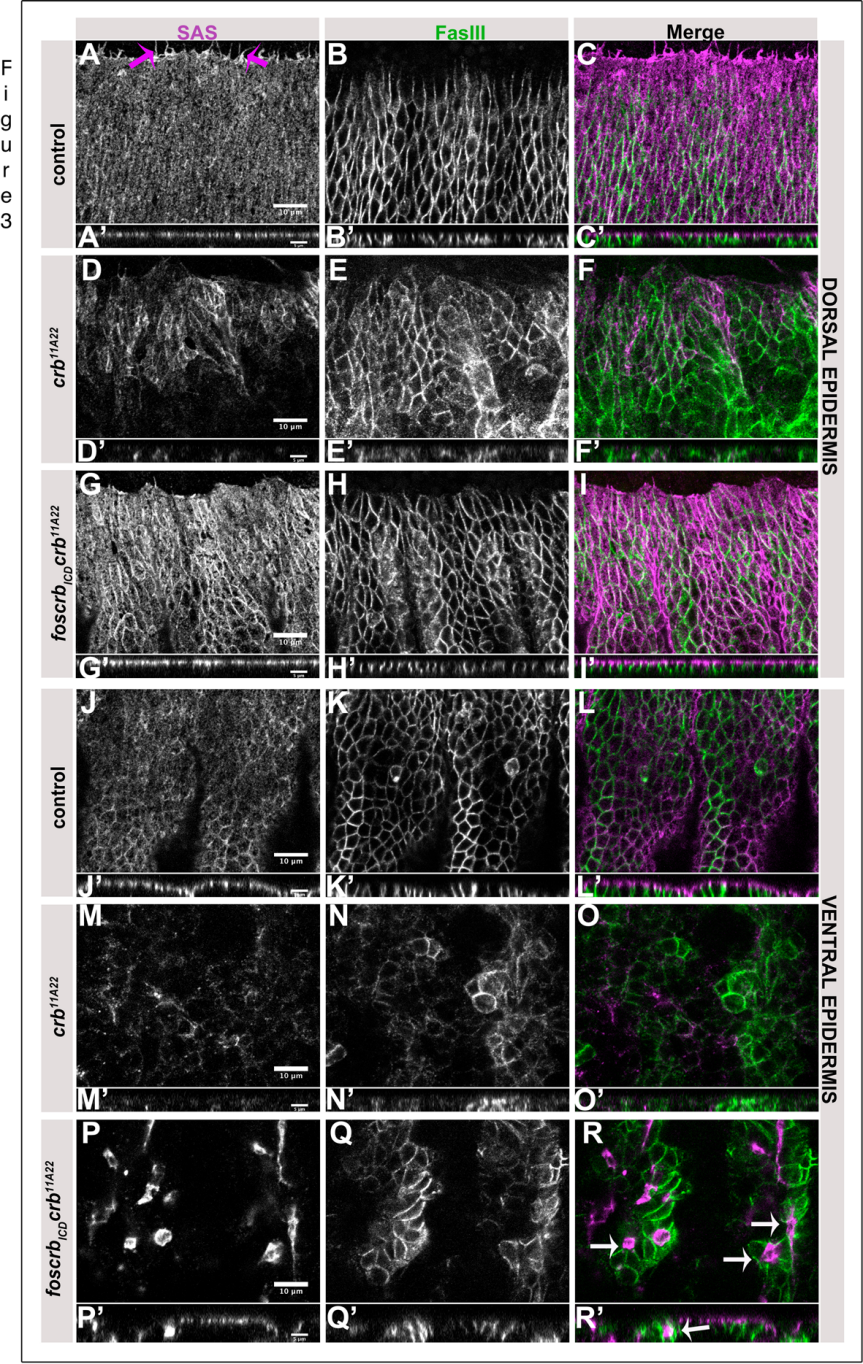
**Apico-basal polarity is restored in *foscrb_ICD_ crb* embryos.** Stage 13 *foscrb; crb^GX24^* control (A-C′,J-L′), *crb^11A22^* (D-F′,M-O′) and *foscrb_ICD_ crb^11A22^* (G-I′,P-R′) embryos stained with anti-SAS (apical) and anti-FasIII (lateral). (A-I,J-R) Lateral view of the dorsal and ventral epidermis respectively. (A′-I′,J′-R′) XZ projections of images in A-I,J-R respectively. Magenta arrows in A point to filopodia. White arrows in R,R′, cyst-like structures with the apical membrane facing the lumen. Scale bar: 10 μm (A-R), 5 μm (A′-R′). The experiment was repeated twice.

Since Crb is required for the maintenance of apico-basal polarity in many embryonic epithelia, we reasoned that the defects observed in the ventral epidermis of *foscrb_ICD_ crb* mutant embryos may be due to incomplete restoration of polarity. Therefore, we scored mutant embryos for the distribution of apical SAS and lateral FasIII, both in the dorsal and in the ventral epidermis (Fig. 3). While the dorsal epidermis of *crb* mutant embryos showed defects in polarity and tissue integrity, the dorsal epidermis of *foscrb_ICD_ crb* embryos developed as a continuous, polarised epithelium, similar as in control embryos (Fig. 3A-I′). Numerous filopodial projections emerged from the dorsal-most epidermal cells of control embryos (magenta arrows in Fig. 3A) (Kaltschmidt et al., 2002). These filopodia were lost in *crb* mutant embryos (Fig. 3D-F), but only incompletely restored in *foscrb_ICD_ crb* mutant embryos (Fig. 3G). The sheet-like organisation of the ventral epidermis was severely disrupted in *crb* mutant embryos, which was associated with a complete loss of apico-basal polarity (compare Fig. 3M-O′ with J-L′). The ICD alone was unable to restore tissue integrity of the ventral epidermis of *crb* mutant embryos: multiple, cyst-like structures were visible, which developed, however, proper apico-basal polarity, with the apical side facing the centre of the cysts (Fig. 3P-R′). From these results, we conclude that cells in the ventral epidermis of *foscrb_ICD_ crb* mutant embryos are polarised, but are unable to maintain a coherent epithelial sheet.

**Fig. 4.**
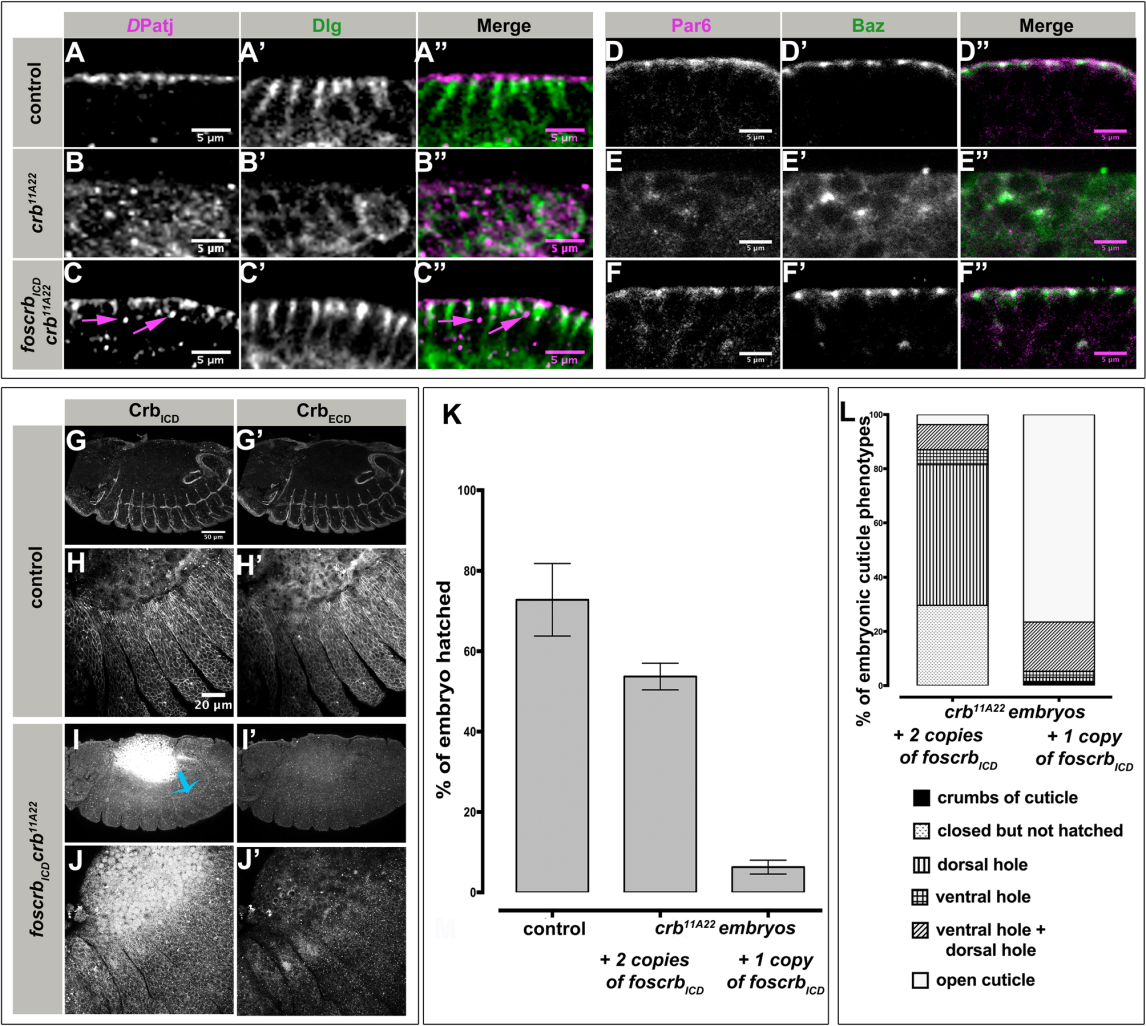
**The Crb ICD partially rescues apico-basal polarity of *crb* mutant embryos.** (A-F″) Stage 12-13 *foscrb; crb^GX24^* control (A-A″,D-D″), *crb^11A22^* (B-B″,E-E″) and *foscrb_ICD_ crb^11A22^* (C-C″,F-F″) embryos, co-stained with anti-*D*Patj (magenta) and anti-Dlg (green) (A-C″) and anti-Par6 (magenta) and anti-Baz (green) (D-F″). Magenta arrows in C and C″, intracellular punctate accumulation of *D*Patj. Scale bar: 5 μm. The experiment was repeated 4 times. (G-J′) Stage 12-13 *foscrb; crb^GX24^* control (G-H′) and *foscrb_ICD_ crb^11A2^2* (I-J′) embryos, co-stained with anti-Crb_ICD_ and anti-Crb_ECD_. H-H′ and J-J′, magnifications of the epidermis shown in G-G′ and I-I′, respectively. Anterior is left, dorsal up. Cyan arrow in I′, hindgut. Scale bar: 50 μm (G,G′,I,I′) and 20 μm (H,H′,J and J′). The experiment was repeated 4 times. (K) Quantification of embryonic lethality. The graph shows the percentage of embryos that hatched. Control: *foscrb; crb^GX24^*. Note that embryos represented by the third column have only one copy of *foscrb_ICD_*. The experiment was repeated 3 times. Error bars show standard error of the mean. (L) Quantification of the cuticle phenotypes of *crb* mutant embryos with one or two copies of *foscrb_ICD_*. The experiment was repeated 3 times.

To further unravel more specifically the restoration of apico-basal polarity in the epidermis of *foscrb_ICD_ crb* mutant embryos, we analysed the localisation of the apical proteins *D*Patj, Par6 and Bazooka (Baz) and the lateral marker Dlg in stage 13 embryos (Fig. 4A-F″). In the epidermis of control embryos, *D*Patj and Dlg are localised in the subapical and the lateral region, respectively (Fig. 4A-A″). The clear segregation of these two proteins was completely lost in *crb* mutant embryos, in that Dlg outlines the whole cell and *D*Patj appeared in intracellular punctae (Fig. 4B-B″). In *foscrb_ICD_ crb* mutant embryos, the segregation of *D*Patj and Dlg into apical and lateral domains is recovered (Fig. 4C-C″), although some punctate staining of *D*Patj inside the cell was still observed (magenta arrows in Fig. 4C,C″). Similarly, the apical and junctional localisation of Par6 and Baz, respectively, was completely lost in *crb* mutant embryos (compare Fig. 4D-D″ and Fig. 4E-E″), but recovered in *foscrb_ICD_ crb* mutant embryos (Fig. 4F-F″). However, only minor amounts of apical Sdt were detected in *foscrb_ICD_ crb* mutant embryos (Fig. S3C-C″, magenta arrows) with punctate staining observed inside the cells (yellow arrow in Fig. S3C). Loss of apical *D*aPKC in epidermal cells of *crb* mutant embryos (compare Fig. S3D and E) was partially restored by *foscrb_ICD_* (Fig. S3F, magenta arrows) but could still be detected intracellularly.

Since enrichment of Crb at the sub-apical region is crucial for localisation of other polarity proteins, and localisation of polarity and junctional proteins was restored in many epithelia upon expression of the ICD, we were interested to know whether the ICD expressed in *foscrb_ICD_ crb* embryos is correctly localised. Therefore, we co-stained embryos with an antibody directed against the ICD of Crb (Crb_ICD_) and an antibody directed against the ECD of Crb (Crb_ECD_). The Crb_ICD_ antibody detects apical Crb protein in all epithelial tissues of control embryos (Fig. 4G), including the epidermis (Fig. 4H) as does the Crb_ECD_ antibody (Fig. 4G′,H′). In contrast, in *foscrb_ICD_ crb* mutant embryos, only minimally apically enriched Crb_ICD_ protein was detected in the epidermis and the trachea (only upon enhancing the contrast) but no apically enriched staining was detected using anti-Crb_ECD_, (Fig. 4I-J′).

Given that we observe only minimal apically enriched Crb_ICD_ but polarity is mostly rescued, we hypothesised that a small amount of apically enriched Crb is sufficient to restore major aspects of polarity. To test this hypothesis, we reduced the copy number of *foscrb_ICD_* by half and analysed the rescue of embryonic lethality. Strikingly, while ∼55% of *crb* mutant embryos with two copies of *foscrb_ICD_* hatched, only 8% of *crb* mutant embryos with only one copy of *foscrb_ICD_* did so (Fig. 4K). Moreover, 80% of the unhatched *crb* mutant embryos with only one copy of *foscrb_ICD_* display a severe cuticle phento (‘open cuticle’ class) (quantified in Fig. 4L), suggesting a widespread failure in maintaining epithelial integrity. In contrast, most of the *crb* mutant embryos with two copies of *foscrb_ICD_* that did not hatch secrete a continuous cuticle with intact denticle belts and head structures (‘closed but not hatched’ category) or develop only a dorsal hole (Fig. 4L and Fig. 1). Together, these results suggest that the ICD of Crb is sufficient to restore apico-basal polarity in a dose-dependent manner, while the ECD is needed to ensure apical enrichment of Crb and hence complete rescue of all aspects of the embryonic *crb* mutant phenotype.

### Loss of Crb or its ECD leads to a neurogenic phenotype due to impaired Notch signalling

**Fig. 5.**
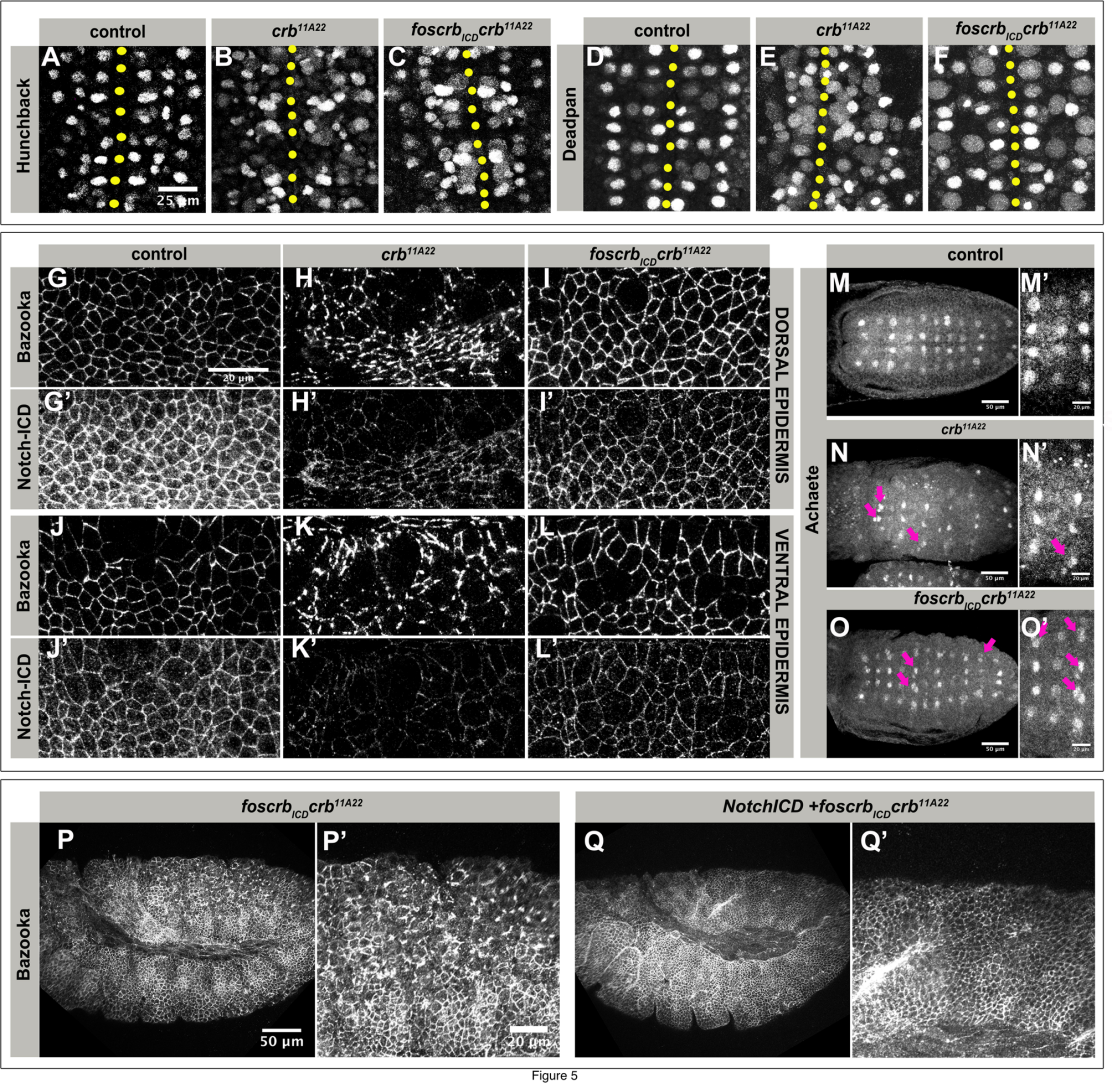
***crb* and *foscrb_ICD_ crb* mutant embryos develop a neurogenic phenotype.** (A-F) Stage 9 *foscrb; crb^GX24^* control (A,D), *crb^11A22^* (B,E) and *foscrb_ICD_ crb^11A22^* (C,F) embryos stained with anti-Hb (A-C) and anti-Dpn (D-F). Yellow dotted line marks the ventral midline. Scale bar: 25 μm. The experiment was repeated 4 times. (G-L′) Dorsal (G-I′) and ventral (J-L′) epidermis of stage 9 *foscrb; crb^GX24^* control (G,G′,J,J′), *crb^11A22^* (H,H′,K,K′) and *foscrb_ICD_ crb^11A22^* (I,I′,L,L′) embryos, stained with anti-Baz and anti-Notch-ICD. Scale bar: 20 μm. The experiment was repeated 3 times. (M-O′) Stage 9 *foscrb; crb^GX24^* control (M,M′), *crb^11A22^* (N,N′) and *foscrb_ICD_ crb*^11A22^ (O,O′) embryos stained with anti-Achaete, anterior to the left. (M′,N′,O′) Close up of images shown in M-O, respectively. Magenta arrowheads in N,N′,O,O′ point to supernumerary neuroblasts. Scale bar: 50 μm (M,N,O) and 20 μm (M′,N′,O′). The experiment was repeated 3 times. (P-Q) Stage 9/10 embryos stained with anti-Bazooka. (P′,Q′) Close up of posterior ventral epidermis of embryos shown in P,Q. Anterior is left. Scale bar: 50 μm (P,Q) and 20 μm (P′-Q′). The experiment was repeated twice.

A more detailed look at the ventral epidermis of *foscrb_ICD_ crb* mutant embryos hinted to defects in neurogenesis. Therefore, we analysed the pattern of neuroblasts by staining for Deadpan (Dpn) and Hunchback (Hb), markers of early neuroblasts (Bier et al., 1992; Jiménez and Campos-Ortega, 1990). *crb* mutant embryos revealed an increase in the number of both Hb- and Dpn-positive cells (compare Fig. 5A and D with Fig. 5B and E, respectively). Expressing *foscrb_ICD_* reduced the number of supernumerary Dpn-positive cells, while no obvious reduction was observed with respect to Hb-positive cells (Fig. 5F and C, respectively). The increased number of neuroblasts observed in *crb* mutant embryos is reminiscent of that observed in neurogenic mutants in which Notch-Delta signalling and hence lateral inhibition is compromised. This leads to specification of more than one neuroblast from a proneural cluster at the expense of epidermoblasts (Hartenstein and Wodarz, 2013). This led us to investigate Notch localisation and signalling in these embryos.

In fact, both *crb* and *foscrb_ICD_ crb* mutant embryos revealed a significant reduction of apical Notch in the dorsal and the ventral epidermis (Fig. 5G-L′, quantified in Fig. S4). To corroborate that Notch activity is weakened in these embryos, we analysed the expression of *achaete* (*ac*), a read-out of Notch activity. *ac* is a proneural gene, expression of which becomes restricted to a single cell within a proneural cluster due to lower Notch signalling in this cell in comparison to neighbouring cells, which experience high Notch activity and hence become specified as epidermoblasts (Skeath and Carroll, 1992) (Fig. 5M,M′). *crb* mutant embryos exhibited less proneural clusters, presumably due to enhanced apoptosis already at this stage. Some of the residual clusters showed more than one Ac-positive cell (magenta arrows in Fig. 5N,N′). *foscrb_ICD_ crb* mutant embryos revealed a pattern of clusters that is similar to that of control embryos. However, in many of these clusters, more than one cell retained expression of Ac (magenta arrows in Fig. 5O,O′).

From these results, we hypothesised that the loss of epithelial integrity observed in *foscrb_ICD_ crb* mutant embryos might be partly due to an increased number of delaminating neuroblasts. Specification of neuroblasts and hence neuroblast delamination can be blocked by expressing the constitutive active intracellular domain of Notch (Notch^intra^) (Lieber et al., 1993; Rebay et al., 1993; Struhl et al., 1993). Expression of Notch^intra^ has been shown to prevent loss of ventral epidermal integrity of *shotgun* (*shg*) mutant or *Cdc42* knock-down embryos (Harris and Tepass, 2008; Tepass et al., 1996). Therefore, we overexpressed Notch^intra^ in *foscrb_ICD_ crb* mutant embryos. The fragmentation of the ventral epidermis was strongly suppressed in these embryos as revealed by the restoration of continuous staining of AJs using Baz (compare Fig. 5P,P′ to Q,Q′).

Taken together, these data suggest that the ECD of Crb is required for apical enrichment of Notch in the embryonic epidermis, and thus ensures proper neuroblast specification via Notch signalling.

### Overexpression of *D*E-Cadherin or Flapwing restores tissue integrity of *foscrb_ICD_ crb* embryos

**Fig. 6.**
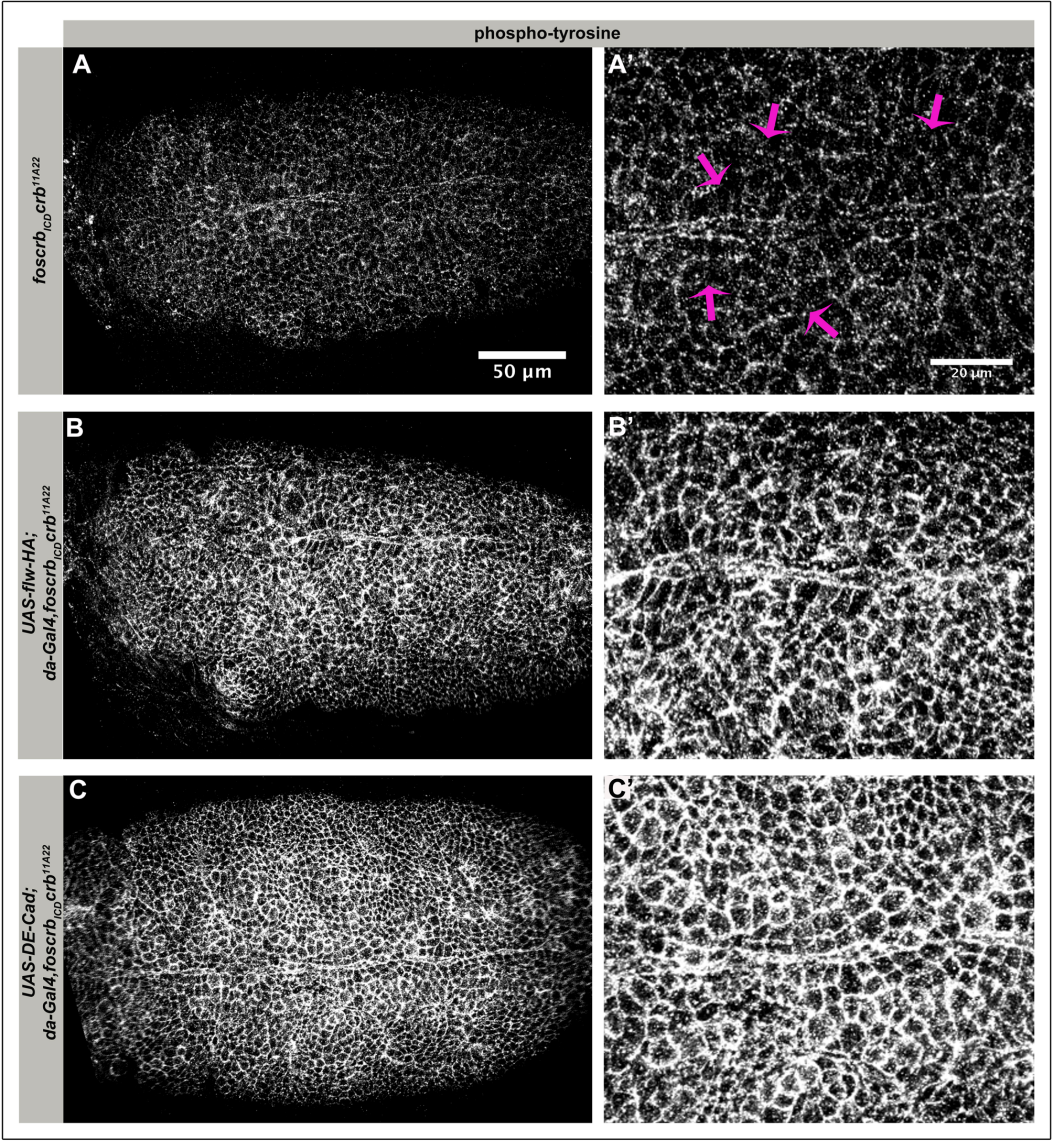
**Defects in the ventral epidermis of *foscrb_ICD_ crb* embryos are rescued by overexpression of *D*E-cadherin or *flapwing*.** Ventral views of stage 9/10 *foscrb_ICD_ crb^11A22^* embryos overexpressing HA-tagged *flapwing* (*flw*; B,B′) or *D*E-cadherin (C,C′), stained with an anti-phosphotyrosine antibody. Yellow lines mark the ventral midline. (A′,B′,C′) Close up of the ventral epidermis of embryos shown in A,B,C, respectively. Magenta arrows in A′ point to cells without proper junctional staining. Anterior to the left. Scale bar: 50 μm (A,B,C) and 20 μm (A′,B′,C′). The experiment was repeated twice.

*foscrb_ICD_* alone could not restore ventral epidermal integrity of *crb* mutant embryos (Fig. 2P), while overexpression of Notch^intra^ in conjunction with *foscrb_ICD_* could. From this we hypothesised that expression of Notch^intra^ not only reduces the number of neuroblasts, but also relieves the ventral epidermis from morphogenetic stress due to reduced neuroblast delamination. It has recently been shown that neuroblast delamination requires increased actomyosin activity (An et al., 2017; Simões et al., 2017). In addition, studies from our lab have shown that Crb negatively regulates myosin contractility in the amnioserosa, thereby ensuring, among others, the maintenance of a proper adhesion belt (Flores-Benitez and Knust, 2015). Therefore, we wondered whether supressing actomyosin contractility or reinforcing AJs in *foscrb_ICD_ crb* mutant embryos could restore ventral epidermal integrity. To address this question, we overexpressed *flapwing* (*flw*) in *foscrb_ICD_ crb* embryos (Fig. 6). *flw* encodes the catalytic subunit of PP1β, a serine/threonine phosphatase, which negatively regulates the myosin regulatory light chain (MRLC), and hence myosin contractility (Vereshchagina et al., 2004). Strikingly, disintegration of the ventral epidermis was suppressed and AJ integrity was restored in these embryos (Fig. 6B,B′). Similarly, overexpressing *D*E-Cadherin in these mutants also rescued epithelial integrity and AJ in the ventral epidermis (Fig. 6C,C′).

Taken together, data presented here show that the ICD of Crb is sufficient to restore apico-basal polarity and integrity of most epithelia of *crb* mutant embryos, while morphogenesis of the neurogenic ectoderm additionally required the ECD. The ECD executes this function, first, by ensuring sufficient apical enrichment of Crb protein, thus stabilising junctions and prevent increased actomyosin activity and second, by stabilising the Notch receptor apically and thus controlling the proper number of delaminating neuroblasts.

## DISCUSSION

By using *foscrb_ICD_* (in the background of a *crb* null allele) we established a genotypic condition similar to a hypomorphic *crb* allele, which enabled us to unveil novel *crb* functions in the *Drosophila* embryo, which are normally hidden by massive polarity defects and apoptosis occurring in the null alleles. Thereby, we gained detailed mechanistic insight into stage- and tissue-specific functions of the ICD and ECD of Crb, which could not be achieved by overexpression studies. We show (i) that a threshold level of the Crb_ICD_ only is sufficient to rescue epithelial cell polarity and even lethality of *crb* mutant embryos. (ii) We provide compelling data for a novel role of the extracellular domain of Crb in embryonic neurogenesis by stabilizing the Notch receptor and thus ensuring proper Notch signaling. (iii) We further show that, in contrast to previous reports, the ECD of Crb is dispensable for the invagination of embryonic epithelial tubes, e.g. the salivary gland.

Earlier structure function analysis of Crb performed in *Drosophila* embryos based on overexpression already suggested an important role of the ICD, since it could rescue polarity defects in many epithelia to the same degree as the full-length protein (Letizia et al., 2013; Wodarz et al., 1995). Therefore, it was suggested that the ICD of Crb is sufficient to perform many Crb functions during embryonic development. Since a fosmid encompassing the whole *crb* locus can completely rescue lethality of *crb* mutant embryos (Klose et al., 2013), we used a similar approach to ask whether the membrane-bound intracellular domain, encoded by a fosmid, has the same rescuing capacity. We provide compelling data to show that the ICD of *Drosophila* Crb is sufficient to rescue lethality of about 50% of homozygous *crb* mutant embryos. It has to be pointed out, yet, that *foscrb_ICD_ crb* mutant animals die as first instar larvae due to various developmental defects, including defects in the maturation of trachea and Malpighian tubules (data not shown).

Furthermore, and in contrast to previous findings based on overexpression studies (Letizia et al., 2013; Röper, 2012), we show that even low amounts of Crb_ICD_ are sufficient to ensure normal invagination of the anlagen of the salivary glands and the tracheae. In addition, the ICD is sufficient to completely rescue AJs and the apical domain in the dorsal epidermis, while the rescue is incomplete in the ventral epidermis. In addition, localization of apical proteins, such as *D*Patj, Par6 and Bazooka/Par3, and the junctional protein *D*E-Cad, is completely restored in the dorsal epidermis. Interestingly, localization of the direct Crb binding partner Sdt was not completely restored under this experimental condition. This could be explained by the low levels of Crb_ICD_ itself, which was hardly detected by immunostainings (and was too low to be detected by western blots; S.D. and E.K., unpublished data). Two reasons can account for this low apical enrichment of Crb_ICD_. (i) Homophilic interactions between the ECDs of Crb molecules have been suggested to stabilise the protein apically in *Drosophila* embryonic and follicle epithelia (Fletcher et al., 2012; Letizia et al., 2013; Röper, 2012), in the primitive streak of early mouse embryos (Ramkumar et al., 2016), and in the zebrafish retina (Zou et al., 2012). Even the short ECD present in mammalian Crb3 was shown to stabilize the protein at the apical membrane, when expressed in GP2-293 cells (Djuric et al., 2016). (ii) Alternatively, the low amount of Crb protein expressed in *foscrb_ICD_ crb* embryos could be explained by reduced trafficking of Crb to the apical membrane in the absence of the ECD. Studies in mouse embryos revealed that O-glycosylation of the EGF-like repeats in the ECD of Crb2 by Protein O-glucosyltransferase 1 (POGLUT1) is essential for proper trafficking to, and enrichment at, the apical membrane. As a consequence, mouse embryos mutant for *POGLUT1* die during gastrulation due to defects in epithelial-mesenchymal transitions (Ramkumar et al., 2015), thus phenocopying defects of embryos lacking *Crb2* (Ramkumar et al., 2016). Replacing all seven putative Rumi/POGLUT1 target sites in *Drosophila* Crb did not affect the viability of homozygous mutant flies (Haltom et al., 2014). This does not, however, exclude a role for other parts of the Crb ECD in apical targeting during embryogenesis. An apical targeting signal may also reside in the cytoplasmic tail of Crb. Targeting of Podocalyxin/Gp135, for example, to the apical membrane of Madine-Darbin-canine kidney (MDCK) cells depends on a bipartite signal, an O-glycosylation-rich region in the ECD and a C-terminal PDZ-domain binding motif in the ICD. During transport of newly synthesized Podocalyxin, EBP50 binds to its PDZ-domain binding motif at the Golgi, thereby inducing its oligomerization and sorting into a clustering complex, which facilitates apical sorting (Yu et al., 2007). It is tempting to speculate that the ICD of Crb may similarly interact with an unknown partner, which directs at least a small amount of Crb_ICD_ to the apical membrane, which is sufficient to rescue cell polarity defects in *crb* mutant embryos.

We are left with the question, how the apical domain of epithelial cells can be formed in the presence of such low levels of apical Crb_ICD_ and Sdt. Previous studies clearly showed that the amount of Crb protein is critical for proper apico-basal polarity. While loss of *Drosophila crb/*mouse *Crb2* results in loss/reduction of the apical surface (Ramkumar et al., 2016; Wodarz et al., 1993, 1995), overexpression of the ICD of *Drosophila* Crb or mammalian Crb3 can lead to an expansion of the apical membrane of epithelial and photoreceptor cells (Klebes and Knust, 2000; Lemmers et al., 2004; Letizia et al., 2013; Muschalik and Knust, 2011; Pellikka et al., 2002; Wodarz et al., 1995). Results presented here suggest that a threshold level of apical Crb, and thus Sdt, is required and sufficient to maintain an apical domain. This assumption is supported by the observation that in the presence of just one copy of *foscrb_ICD_* only 8% of *crb* mutant embryos hatch, compared to 50% in the presence of two copies of *foscrb_ICD_*, while one copy of *foscrb*, which encodes full-length Crb proteins, is sufficient to fully rescue lethality of *crb* mutant embryos (Klose et al., 2013). Unlike Sdt, *D*aPKC was apically enriched in *foscrb_ICD_ crb* embryos, but was also detected within the cell. Removal of one copy of endogenous *D*aPKC in *foscrb_ICD_ crb* embryos enhanced embryonic lethality (S.D. and E.K.,unpublished data), making it unlikely that the phenotypes observed *in foscrb_ICD_ crb* embryos are due to increased phosphorylation of the Crb_ICD_ as a result of upregulation of *D*aPKC. Although phosphorylation of two threonine residues in Crb_ICD_ by aPKC was suggested to be functionally important (Sotillos et al., 2004), recent results showed that mutation of these residues to non-phosphorylatable alanine have no effect on viability and fertility of homozygous mutant flies (Cao et al., 2017). Interestingly, expression of a stable form of *D*E-cadherin can restore AJ formation and polarity in embryonic epithelia even in the absence of *sdt* or *crb* (Chen et al., 2017), suggesting other, Crb complex-independent mechanisms to ensure apico-basal polarity. Further investigations on the relationship between *sdt*, *D*aPKC and Crb_ICD_ are needed to completely understand the significance of the upregulation of *D*aPKC observed and its possible effect on embryonic epidermal integrity.

*crb* mutant embryos expressing two copies of *foscrb_ICD_*, which fail to hatch, develop defects in the amnioserosa and the ventral epidermis, two tissues exhibiting a high degree of morphogenetic activity, which is in line with earlier proposals suggesting that Crb/the Crb-complex is particularly required in dynamic epithelia with high turnover of AJs (Campbell et al., 2009; Chen et al., 2017). The disruption of the mono-layered organisation of the ventral epidermis of *foscrb_ICD_ crb* mutant embryos goes along with the formation of ‘cyst’-like structures, probably due to weakened AJs. Similar defects in the ventral epidermis were observed in embryos in which *Cdc42* was knocked down (Harris and Tepass, 2008) and in *shotgun* (*shg*) mutant embryos, which lack the gene encoding *D*E-cadherin (Tepass et al., 1996; Uemura et al., 1996). We would like to point out an important difference observed in the phenotypes of the ventral epidermis in *foscrb_ICD_ crb* embryos and in *shg* mutant embryos: while in both mutants AJs fail to be maintained, *foscrb_ICD_ crb* mutant embryos additionally show an increased number of neuroblasts, as revealed by an increased number of Hunchback (Hb)-positive cells, while *shg* mutant embryos do not show any defect in neuroblast numbers (Wang et al., 2004). Neuroblasts are the precursors of the ventral nerve cord, which delaminate from the ventral neurogenic ectoderm (Hartenstein and Campos-Ortega, 1984). Neuroblast delamination is characterised by an anisotropic loss of AJs, apical constriction due to periodic myosin pulsation, followed by the gradual disappearance of the apical membrane (An et al., 2017; Simões et al., 2017). Once neuroblasts have delaminated, the remaining cells within the epithelium have to close the gap by reforming AJs. Neuroblast number and spacing is controlled by the Notch signalling pathway, and loss of any of the neurogenic genes, which encode constituents of this pathway, results in a hyperplasia of the nervous system at the expense of the epidermis (Campos-Ortega and Knust, 1990; Hartenstein and Wodarz, 2013; Lehmann et al., 1981).

Defects in two, mutually not exclusive, mechanisms may account for the phenotype in the ventral epidermis associated with reduction of *crb*, namely reduced Notch signalling and enhanced uncontrolled actomyosin activity. First, reduced Notch levels observed in the absence of Crb_ICD_ lead to reduced Notch signalling, and consequently, to impaired lateral inhibition and an increased number of neuroblasts, which create additional stress during delamination. In fact, expression of the intracellular, constitutively active form of Notch, Notch^intra^, was able to suppress the disintegration of the ventral epidermis of *foscrb_ICD_ crb* mutant embryos. Two scenarios could account for impaired Notch signalling in the ventral neurogenic ectoderm in the absence of the ECD of Crb. (i) Reduced apical Notch protein in *crb* and in *crb fos_ICD_* embryos due to impaired stabilisation by Crb or Crb_ECD_ results in reduced Notch signalling in the neuroectoderm. Similar observations were made in the developing dorsal telencephalon of *Crb2* mutant mice, which is associated with premature expression of differentiation genes and an increase in basal neural progenitor cells at the cost of the apical progenitor pool (Dudok et al., 2016). This phenotype has striking similarity to that induced upon inactivation of *Notch1*, which is characterized by the loss of progenitor pools and premature neural differentiation (Mizutani et al., 2007). In other cases, loss/reduction of Crb can result in activation of the Notch pathway. In the developing pupal wing, depletion of Notch from the apical surface in the absence of Crb provokes the activation of the ligand-independent Notch pathway, followed by cell fate specification defects (Nemetschke and Knust, 2016). Similarly, zebrafish Crb was shown to interact with Notch when expressed in cell culture, and overexpression of Crb reduced Notch activity (Ohata et al., 2011). (ii) Alternatively, the absence or reduction of the Crb ECD may affect the Notch signalling pathway indirectly due to effects on apico-basal polarity, since in many cells receptors are enriched and activated at the apical pole. In fact, in zebrafish, an apico-basal gradient of Notch is instrumental for ensuring apical mitosis and proper cell fate decision, both in the neuroepithelium and the retinal epithelium. Expansion of the apical surface upon loss of Lgl1 (Clark et al., 2012) results in increased apical Notch activity, which prevents premature differentiation. To discriminate between these two possibilities, replacing the Crb ECD by a heterologous, extracellular dimerization domain may stabilise the Crb_ICD_ and thus lead to normal levels of apical Crb, but would not be able to stabilise Notch.

Beside impaired Notch signalling and hence increased neuroblast delamination, our data support the conclusion that uncontrolled actomyosin activity contributes to the phenotype observed in *foscrb_ICD_ crb* mutant embryos. Coupling between actomyosin and AJ is essential for epithelial stability, and enhanced tensile forces due to increased actomyosin activity can be detrimental for AJ stability and epithelial integrity (Arnold et al., 2017; Citi et al., 2014; Heisenberg and Bellaïche, 2013). We previously showed that a mutation in the FERM-domain binding motif of Crb induces increased actomyosin activity in the amnioserosa, followed by severe disintegration of the epithelium and defects in dorsal closure. This defect could be rescued by overexpression of *flapwing* (*flw*) (Flores-Benitez and Knust, 2015). Based on the observation that overexpressing *flw* suppresses the disintegration of the ventral epidermis in *foscrb_ICD_ crb* mutant embryos as well, it is tempting to speculate that insufficient apical enrichment of Crb_ICD_ contributes to increased actomyosin activity also in the neuroectoderm. So, either reducing actomyosin activity by overexpression of *flw* or preventing neuroblast delamination by expressing Notch^intra^ and thereby activating the Notch pathway can release excess morphogenetic stress in the neurogenic ectoderm of *foscrb_ICD_ crb* mutant embryos. Detailed measurements of actomyosin activity upon loss or reduction of Crb are needed to achieve further mechanistic insight into the role of Crb in actomyosin-mediated tension in the neuroectoderm.

In summary, the approach used here allowed us to systematically dissect the tissue-specific roles of different domains of the Crb protein during *Drosophila* embryogenesis. In the neurogenic ectoderm, the ECD of Crb is not only required to counteract increased tension due to neuroblast delamination, but also to ensure proper Notch signalling and thereby control the number of neuroblasts. Further studies will reveal how the activities of the different Crb protein domains are coordinated to ensure tissue homeostasis in different epithelia.

## MATERIALS AND METHODS

### Recombineering for generation of constructs

An improved counter-selection strategy that used the pABRG as the helper plasmid and phosphothioated oligonucleotides as ‘modification cassettes’ were used to generate the foscrb variants in this study (Bird et al., 2011). Single stranded oligonucleotides, with two 5′ phosphothioate bonds, that either target the endogenous lagging strand or the leading strand were used. A list of the ‘modification rpsl-neo cassettes’, the counter-selection oligonucleotides is mentioned in the supplementary Materials and Methods. All the exons in the newly created transgenic constructs were sequenced. A detailed, step-by-step description of the protocol used is available on request.

### Generation of transgenic flies

Transgenic flies were generated with the help of phiC31 integrase mediated site-specific integration into VK00033 landing site y^1^, w*, P{nos-phiC31int.NLS}X; PBac{y+-attP-3B}VK00033 (BL-32542) (Sarov et al., 2016). Correct transformants were screened for red fluorescent eyes (from 3XP3-Dsred marker in fosmid backbone) (Ejsmont et al., 2009).

### Fly stocks

Flies were maintained on standard food at 25°C. For most of the experiments, *crb* mutant alleles were balanced over fluorescent balancers to distinguish the homozygous mutant embryos from the rest of the embryos. In cases where the mutant flies could not be maintained over the fluorescent balancers, they were maintained over non-fluorescent balancers and the homozygous mutant embryos were distinguished by staining for Crb. All experiments were performed using the protein-null alleles *crb^11A22^* or *crb^GX24^*, thus ensuring that the different versions of *crb* is expressed only from the fosmid. All fly stocks are listed in supplementary Materials and Methods. *Drosophila* manipulations were done in accordance with standard techniques.

### Embryo collection and antibody staining

Embryos were collected in fly cages on apple juice agar plates at 25°C. For most experiments, 2 h or 1 h collections were done. Staging was based on control embryos (*foscrb;crb^GX24^*). Embryos of different genotypes used in the same experiment were collected and aged for the same time and fixed and stained in parallel. Embryos were dechorionated with 3% sodium hypchlorite (3 min) and fixed in 4% formaldehyde in phosphate-buffered saline (PBS)/heptane with a V/V of 1:1 on a rotating shaker (25 min). Embryos were devitellinized in a solution of heptane/methanol (1:1). For Achaete antibody staining, PEM buffer was used (4 ml PEM, 1 ml FA 37%, 5 ml heptane) and embryos were fixed for 20 min. For staining with Sdt-, Bazooka- and Notch-Intra antibodies, heat fixation of embryos was used (Müller, 2008). Fixed embryos were washed thrice and stored in 100% methanol at −20°C for future use. For staining, embryos were gradually rehydrated at room temperature in decreasing concentration of methanol (75%, 50%, 25%, 0%) in PBT (0.3 Tx-100). Embryos were incubated in blocking solution with 5% normal horse serum (NHS; Sigma-Aldrich H1270, St. Louis, Missouri, USA) in PBT (0.3 Tx-100) for two hours, followed by an overnight incubation with primary antibodies diluted in 5% NHS containing PBT (0.3Tx-100). The embryos were washed in PBT (0.3% Tx-100) four times for 15 min each and then incubated in the appropriate secondary antibodies (Alexa conjugated) diluted in 5% NHS/PBT (0.3Tx-100) for two hours. Embryos were washed in PMT (0.3% Tx-100) and mounted on glass slides using VectaShield. Homozygous *crb* mutant embryos were preselected prior to fixing by selecting against the GFP signal from the fluorescent balancers under a dissecting microscope. To identify *crb* mutant embryos at stage 9/10, embryos were stained with anti-Crb antibody and only those embryos without the Crb signal were imaged. Images were acquired using a Zeiss LSM 780 NLO confocal microscopy (ZEISS Microscopy) with a C-Apochromat 40×/1.2W Corr objective. Image analysis and pseudocolor assignment were done in Fiji and images were assembled in Adobe Photoshop CC. Image manipulation was fully compliant with the image guidelines for proper digital image handling outlined in Rossner and Yamada (2004).

### Cuticle preparation

Cuticle preparations were performed as described recently (Flores-Benitez and Knust, 2015). Images of the cuticle were acquired by phase contrast with Zeiss Axio Imager.Z1 microscope with an EC Plan-NEOFLUAR 10× objective. Images were visualized and modified in Fiji and assembled in Adobe Photoshop.

### Live imaging of embryos

Life imaging of embryos was essentially performed as described recently (Flores-Benitez and Knust, 2015). Embryos were imaged by multi-position and multi-time scanning using a Zeiss LSM 780 NLO confocal microscope with a W Plan-Apochormat 40×/1.0 objective. 4-D hyperstakcs were processed with Fiji. Image manipulation was fully compliant with the image guidelines for proper digital image handling outlined in Rossner and Yamada (2004).

### Statistical analysis

Graphs were plotted and the statistical analyses were performed using GraphPad Prism6. Results are expressed as means±s.d. Statistical significance was calculated by an unpaired Kolmogorov–Smirnov test (Fig. S4).

## Supplementary Material

Supplementary information
