# Supplementary material for: A dual role of the extracellular domain of Drosophila Crumbs for morphogenesis of the embryonic neuroectoderm
Source: Biol Open. 2018 Jan 15;7(1):bio031435. doi: 10.1242/bio.031435 (PMC5829512; doi:10.1242/bio.031435)
Supplement: Supplementary information [file biolopen-7-031435-s1.pdf]

## Supplementary figures and movies

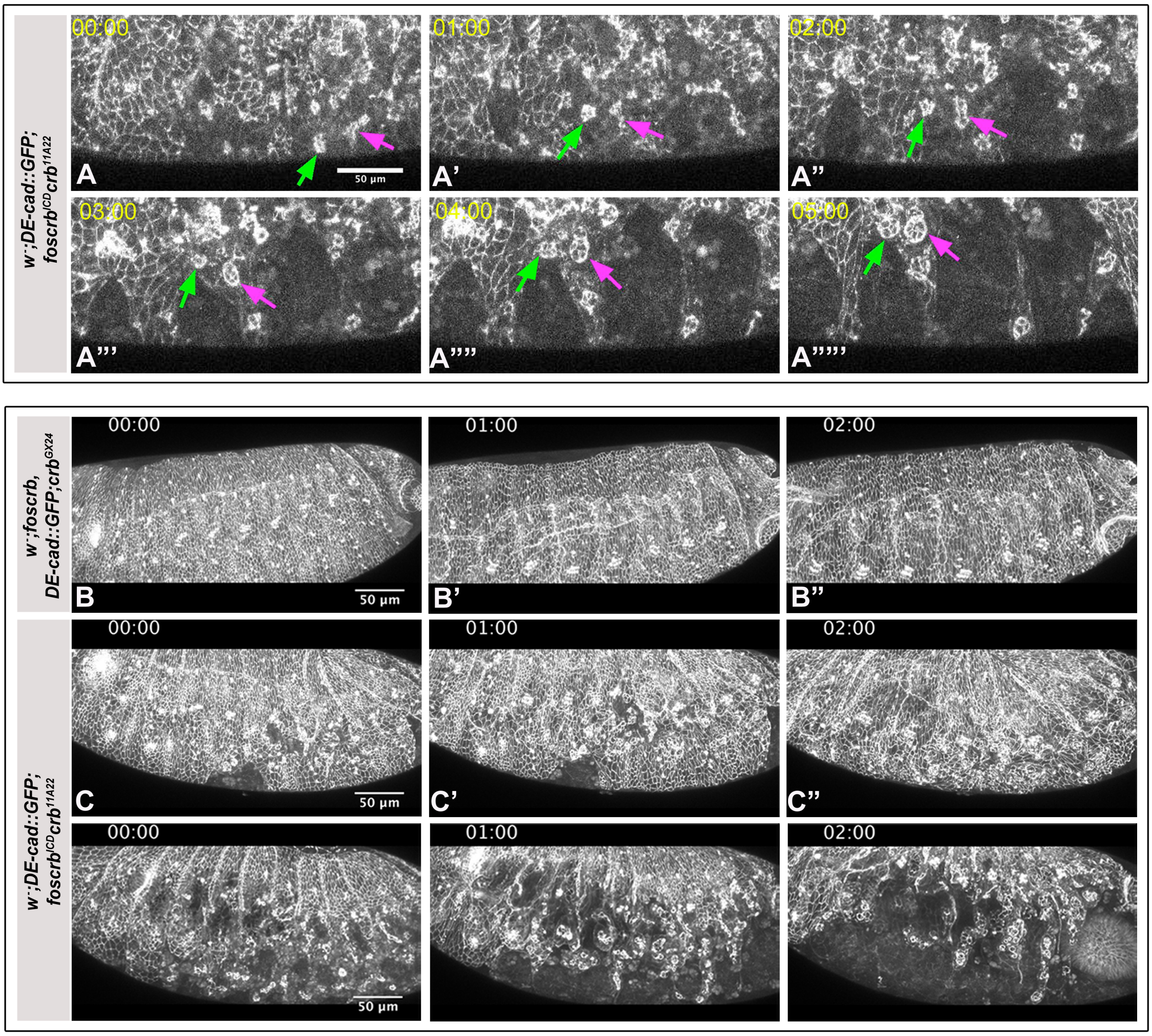


**Supplementary Figure S1.** (For Fig.2)

**A-A'''''**: Higher magnifications of stills of time lapse movies shown in Fig. 3, to highlight the phenotypes in the ventral epidermis. Magenta and green arrows point to two different groups of cells that gradually assume a "cyst-like" structure. Scale bar = 50µm.

**B-D''**. Stills of time lapse movies showing expression of GFP-tagged *D*E-Cadherin in embryos after dorsal closure. Control: *foscrb, DE-cad;;GFP; crb*$^{GX24}$. C-C" and D-D" show two different embryos of the same genotype. Anterior is left, dorsal up. Scale bar = 50µm.

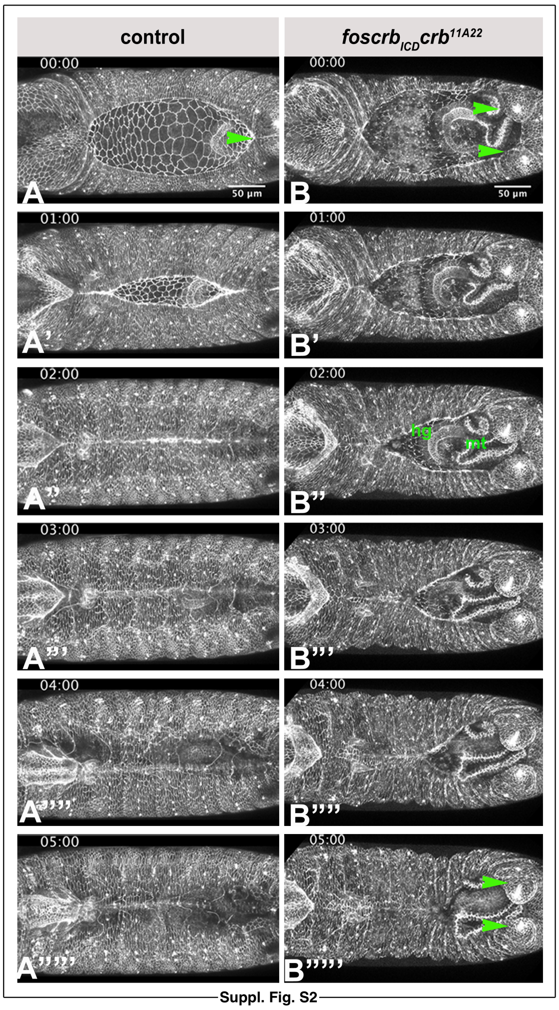


Suppl. Fig. S2

**Supplementary Figure S2.** (For Fig.2)

**A-B”””:** Stills of time lapse movies showing expression of GFP-tagged *D*E-Cadherin in embryos during dorsal closure. Control: *foscrb, DE-cad;;GFP; crb*$^{GX24}$. Dorsal closure fails in some mutant embryos with hg and Mt exposed out (B”). The posterior zippering is not initiated due to failure in fusion of the posterior most abdominal segments (green arrow heads in B and B”””). Scale bar = 50µm.

Biology Open • Supplementary information

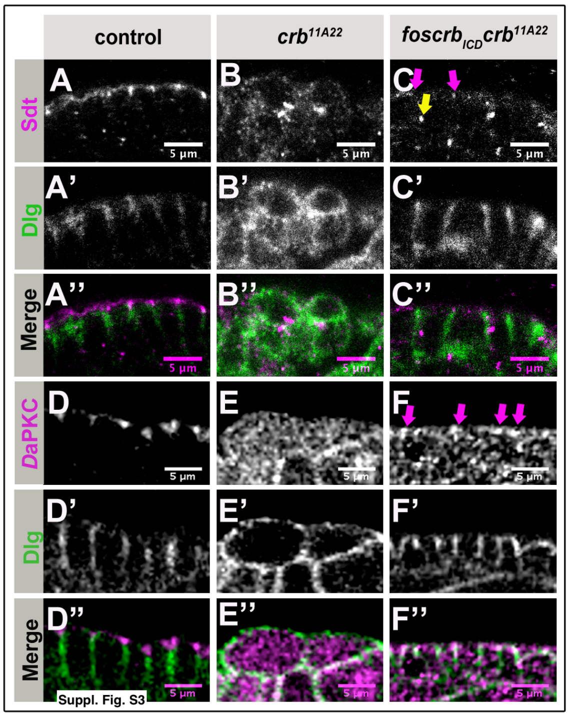


**Supplementary Figure S3.** (For Fig.4)

Stage 13 *foscrb; crb*$^{GX24}$ control (A-A'', D-D''), *crb*$^{11A22}$ (B-B'', E-E'') and *foscrb*$_{ICD}$ *crb*$^{11A22}$ (C-C'', F-F'') embryos stained with either Sdt or *D*aPKC (apical) and Dlg as the (lateral). Magenta arrow in C: apical enrichment of Sdt; yellow arrow in C: punctae staining of Sdt inside cells; magenta arrow in F: Apical enrichment of *D*aPKC. Scale bar = 5 µm

Biology Open • Supplementary information

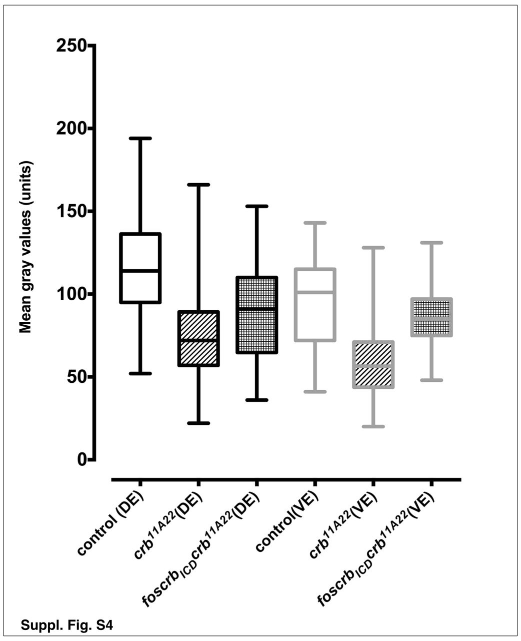


**Supplementary Figure S4.** (For Fig.5)

Quantification of Notch intra staining. For the imaging, a z-stack was imaged in similar areas for controls and mutants in the dorsal epidermis and ventral epidermis. The slice with apical Bazooka staining was used to quantify the intensities for Notch Intra staining ensuring mostly apical Notch enrichment was quantified.

Biology Open • Supplementary information

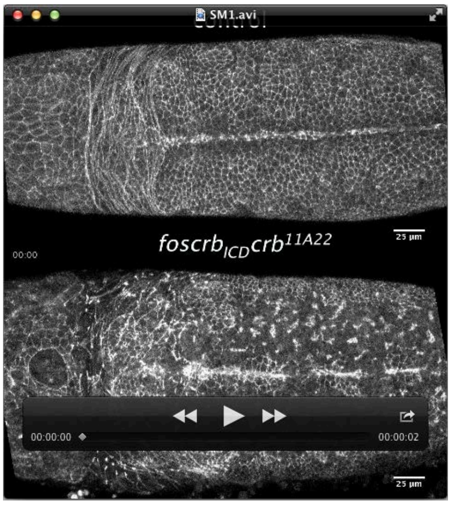


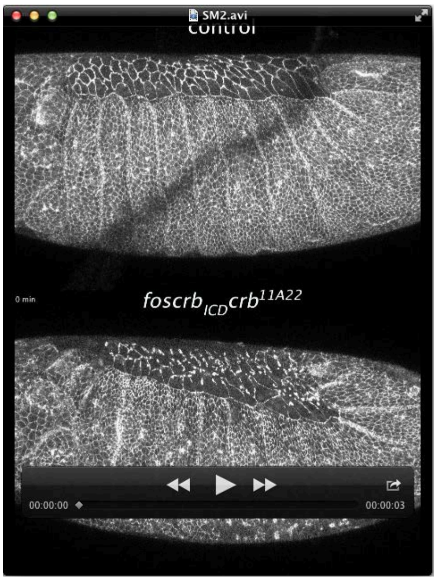


**Supplementary Movie M1, M2** (For Fig.2)

Time lapse movies of endogenously tagged *D*E-Cadherin-GFP lines during germ band retraction (dorsal view) (M1) and dorsal closure (lateral view)(M2).

Biology Open • Supplementary information

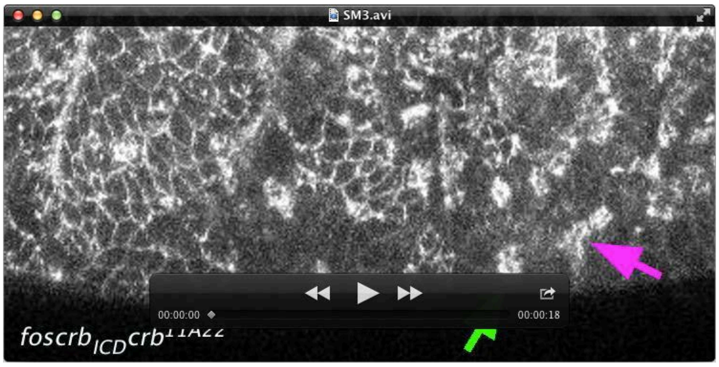


**Supplementary Movie M3** (For Fig.2)

Close up of the disintegrated ventral epidermis of the *foscrb*$_{ICD}$ *crb*$^{11A22}$ embryos from M2.

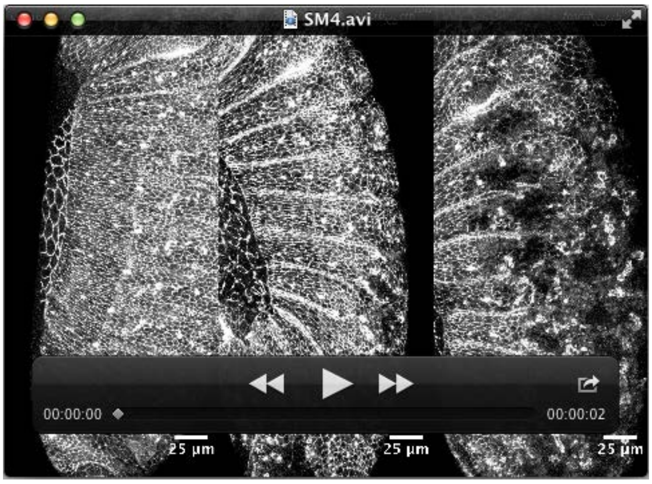


**Supplementary Movie M4** (For Fig.2)

Two embryos of the genotype *foscrb*$_{ICD}$ *crb*$^{11A22}$ tagged with *D*E-Cadherin-GFP showing different degrees of rescue.

Biology Open • Supplementary information

# Supplementary Material and Methods

## Establishment of transgenic fly lines

The procedure is modified from that published previously (Bird et al., 2011)

Region of *crb* where homology arms were targeted:

A description of the different regions in the *crb* locus, where the forward and reverse homology arms were targeted for the *foscrb* variants is mentioned below. The introns are represented in small letters (in black) and the exons are represented in capital letters (in blue). The region of the homology arm is highlighted in green and the stop codon TAG is mentioned in bold letters. The corresponding exon no. is mentioned on the right.

a) *foscrb*$_{ICD}$:

Forward homology arm

| | |
|---|---|
| tgcagATGTCGCCTCAGTGGCGGTGCCGACGAAGGAGGCGTACTTTAATGGCTCCA<br>CTTACCTCCGCCTCACCACGCCGATGCCCATTTGGGATCACTCGGCGATTAGTTT<br>CCGCTCGTGCCGCGGCGGCGAGATCCTCGCCCAGCAGTACAACAAGAACTCCAT<br>TGTAATCTCAGTGCTCAATGACTTTCTGCAAATCTCACTGGCTGGACCCGCCGTC<br>CATGGGCCCAACAACCGGCTGGATGTCAAGCTGCCCTACCAACTGCTGGACAAC<br>CGCTGGCATACGCTGCAGTTCAAGTACGAGTACGGAAATCTCTACCTGCATGTG<br>GATCGCGCGGCAAGCATATTTGgtaagtaaccatgccgttctggctgccccaaattggatttcaggctctatcgc<br>ccactcattcgggtcag | Exon 2 |

Reverse homology arm

| | |
|---|---|
| GTACCGATGTGCAAATGTAGTCTGGGATACACTGGTCGCCTGTGCGAGCAGGAC<br>ATTAACGAGTGCGAATCGAATCCATGCCAGAACGGTGGTCAGTGTAAGGACCTC<br>GTCGGCAGGTACGAGTGCGATTGCCAGGGCACCGGGTTCGAGGGCATTCGCTGT<br>GAAAATGACATCGACGAGTGCAACATGGAGGGAGATTACTGCGGCGGATTGGG<br>CCGGTGTTTCAACAAGCCCGGATCCTTCCAGTGCATCTGCCAGAAACCCTATTG<br>CGGAGCCTACTGCAACTTTACGGATCCCTGCAACGCTACGGACCTCTGCTCAAA<br>CGGCGGTCGCTGCGTAGAGTCCTGCGGCGCCAAACCGGACTACTACTGCGAGTG<br>TCCGGAAGGTTTCGCGGGAAAGAATTGCACAGCACCGgtaagttttcaatgatacactacaaattc<br>agtgaaagaaaaacaatatattgatttcttcgctttttttcagATTACGGCCAAGGAGGACGGGCCTTCGACC<br>ACAGACATTGCCATCATTGTAATACCCGTAGTGGTGGTGCTGCTGCTGATCGGG<br>GAGCCCTCCTGGGCACCTTCCTGGTGATGGCCAGGAACAAGCGAGCAACCAGG<br>GGCACCTATAGCCCGAGCGCGCAAGAGTACTGCAACCCACGGCTGGAAATGGA<br>CAACGTACTGAAGCCACCGCCGGAAGAGCGACTAATT**TAG…………** | Exon 12<br><br><br><br><br><br><br><br><br>Exon 13 |

Biology Open • Supplementary information

b) *foscrb*$_{EGFP\text{-}ECD}$

Forward homology arm: The two sequences shaded in green were combined to generate the forward homology arm. This was done to make sure that the stop codon (bold TAG) is in frame.

| | |
|---|---|
| gtaagttttcaaatgatacactacaaattcagtgaaagaaaaacaatatattgatttcttcgctttttcagATTACGGCCA AGGAGGACGGGCCTTCGACCACAGACATTGCCATCATTGTAATACCCGTAG TGGTGGTGCTGCTGCTGATCGCGGGAGCCCTCCTGGGCACCTTCCTGGTGAT GGCCAGGAACAAGCGAGCAACCAGGGGCACCTATAGCCCGAGCGCGCAAG AGTACTGCAACCCACGGCTGGAAATGGACAACGTACTGAAGCCACCGCCGG AAGAGCGACTAATTTAGTTTTGAGTTTTGAGCA…… | Exon 13 |

Reverse homology arm

| | |
|---|---|
| ATTACGGCCAAGGAGGACGGGCCTTCGACCACAGACATTGCCATCATTGTA ATACCCGTAGTGGTGGTGCTGCTGCTGATCGCGGGAGCCCTCCTGGGCACCT TCCTGGTGATGGCCAGGAACAAGCGAGCAACCAGGGGCACCTATAGCCCGA GCGCGCAAGAGTACTGCAACCCACGGCTGGAAATGGACAACGTACTGAAG CCACCGCCGGAAGAGCGACTAATT**TAG**TTTTGAGTTTTGAGCATGAACGAC GATTAGCAAAGCAAACAAAAGATATTTTTAAATCCGCCCATATACACCTAG CTGTAGGAGTAACTCAATGTTTTGTACTAAGTT…… | Exon 13 |

<u>The homology arms used</u>

The sequences of the homology arms used to generate and amplify the rpsl-neo modification cassette are given below. The homology arms consist of a sequence corresponding to *crb* locus (in blue letters) followed by a sequence corresponding to the rpsl-neo casstte (in black letters).

| **foscrb variant** | **Forward homology arm** | **Reverse homology arm** |
|---|---|---|
| foscrb$_{ICD}$ | ATGTCGCCTCAGTGGCGGTG CCGACGAAGGAGGCGTACT TTAATGGCTCCGGCCTGGTG ATGATGGCGGGA | GTTTTTCTTTCACTGAATTTG TAGTGTATCATTTGAAAACT TACCGGTGCTCAGAAGAACT CGTCAAGAAGG |
| foscrb$_{EGFP\text{-}ECD}$ | CCCTCCTGGGCACCTTCCTG GTGATGGCCAGGAACAAGC GAGCAACCAGGATTTAGGG CCTGGTGATGATGGCGGGAT CG | AATATCTTTTGTTTGCTTTGC TAATCGTCGTTCATGCTCAA AACTCAAAATCAGAAGAAC TCGTCAAGAAGG |

Table 1 : Homology arms used for the first recombineering step

Biology Open • Supplementary information

List of oligonucleotides used for the 2nd recombineering step

| foscrb variant | Lagging strand | Leading strand |
|---|---|---|
| $foscrb_{ICD}$ | A*T*GTCGCCTCAGTGGCGGTGCCGACGAAGGAGGCGTACTTTAATGGCTCCGCACCGGTAAGTTTTCAAATGATACACTACAAATTCAGTGAAAGAAAAAC | G*T*TTTTCTTTCACTGAATTTGTAGTGTATCATTTGAAAACTTACCGGTGCGGAGCCATTAAAGTACGCCTCCTTCGTCGGCACCGCCACTGAGGCGACAT |
| $foscrb_{EGFP\text{-}ECD}$ | C*C*CTCCTGGGCACCTTCCTGGTGATGGCCAGGAACAAGCGAGCAACCAGGATTTAGTTTTGAGTTTTGAGCATGAACGACGATTAGCAAAGCAAACAAAAGATATT | A*A*TATCTTTTGTTTGCTTTGCTAATCGTCGTTCATGCTCAAAACTCAAAACTAAATCCTGGTTGCTCGCTTGTTCCTGGCCATCACCAGGAAGGTGCCCAGGAGGG |

Table 2: Single stranded oligos used in the second recombineering step

List of primers used for PCR and sequencing

| foscrb variant | Forward primer | Reverse primer |
|---|---|---|
| $foscrb_{ICD}$ | GGCCTGGTGATGATGGCGGGATCG | TACGCTTAGATACTCTAGAGC |
| $foscrb_{EGFP\text{-}ECD}$ | ACCTGCCAGAATGGATTCA | TCAGAAGAACTCGTCAAGAAGG |

Table 3: Primers for checking correct integration of rpsl-neo cassette

| foscrb variant | Forward primer | Reverse primer |
|---|---|---|
| $foscrb_{ICD}$ | GGCAATCTTGATCTTCTCTTG | TACGCTTAGATACTCTAGAGC |
| $foscrb_{EGFP\text{-}ECD}$ | GGTACGAGTGCGATTGCCAG | TACGCTTAGATACTCTAGAGC |

Table 4: Primers for checking correct removal of rpsl-neo cassette

| foscrb variant | Forward primer | Reverse primer |
|---|---|---|
| $foscrb_{ICD}$ | GGCAATCTTGATCTTCTCTTG | TACGCTTAGATACTCTAGAGC |
| $foscrb_{EGFP\text{-}ECD}$ | GGTACGAGTGCGATTGCCAG | TACGCTTAGATACTCTAGAGC |

Table 5: Primers for sequencing generated variants

## List of fly lines and antibodies used in this study

| | |
|---|---|
| *w*$^{-}$*;;crb*$^{11A22}$*/TTG* | *crb* null allele; BSC #3448 |
| *w*$^{-}$*;;crb*$^{GX24}$*/TTG* | *crb* null allele (Huang et al., 2009) |
| *w*$^{-}$*; foscrb;crb*$^{GX24}$ | (Klose et al., 2013) |
| *w*$^{-}$*;;foscrb*$_{ICD}$*crb*$^{11A22}$*/TTG* | This study |
| *w*$^{-}$*;;foscrb*$_{ICD}$*crb*$^{GX24}$*/TTG* | This study |
| *w*$^{-}$*;;foscrb*$_{EGPF\text{-}ECD}$*crb*$^{GX24}$*/TTG* | This study |
| *w*$^{-}$*;;foscrb*$^{EGFP}$*crb*$^{GX24}$ | This study |
| *w*$^{-}$*;DE-cad::GFP* | *D*E-cadherin fused with GFP knock-in allele; homozygous viable (Huang et al., 2009) |
| *w*$^{-}$*;UAS-flw-HA* | BSC #23703 |
| *w-;UAS – Notch[intraMHLS]/Cyo[wg-lacZ];MKRS/TM6* | Gift from Thomas Klein (Klein and Arias, 1998) |
| *UAS-DE-cad* | Klebes, A. and Knust, E. (unpublished results) |
| *TTG* | BSC #6663 |

Table 6: Fly lines used in this study

| | Animal | Dilution | Source |
|---|---|---|---|
| anti-Crb2.8 | Rat | 1:500 | (Richard et al., 2006) |
| anti-Crb Intra | Rabbit | 1:200 | (Kumichel et al., 2015) |
| anti-Disc Large | Mouse | 1:50 | DSHB #4F3 |
| anti-Bazooka | Rabbit | 1:500 | (Wodarz et al., 1999) |
| anti-*D*Patj | Rabbit | 1:1000 | (Richard et al., 2006) |
| anti-Stranded at Second | Rabbit | 1:500 | (Wodarz et al., 1995). Kindly provided by D.Cavener |
| anti-Hunchback | Guinea pig | 1:500 | (Mettler et al., 2006). Kindly provided by J.Urban |
| anti-Stardust | Rabbit | 1:500 | (Berger et al., 2007) |
| anti-Par6 | Guinea pig | 1:500 | (Kim et al., 2009). Kindly provided by A.Wodarz |
| anti-aPKC | Rabbit | 1:200 | Santa Cruz C20 |
| anti-Notch | Mouse | 1:100 | DSHB C17.9C6 Conc. Purified |
| anti-Neurotactin | Mouse | 1:10 | DSHB BP106 |
| anti-Achaete | Mouse | 1:10 | (Skeath and Carroll, 1992). DSHB |
| anti-Deadpan | Guinea pig | 1:500 | (Levy and Larsen, 2013). Kindly provided by J. Skeath |
| anti-phospho tyrosine | Mouse | 1:100 | BD Biosciences |

Table 7: List of primary antibodies used in this study

## References


Berger, S., N.A. Bulgakova, F. Grawe, K. Johnson, and E. Knust. 2007. Unravelling the genetic complexity of *Drosophila stardust* during photoreceptor morphogenesis and prevention of light-induced degeneration. *Genetics*. 176:2189-2200.

Bird, A.W., A. Erler, J. Fu, J.K. Heriche, M. Maresca, Y. Zhang, A.A. Hyman, and A.F. Stewart. 2011. High-efficiency counterselection recombineering for site-directed mutagenesis in bacterial artificial chromosomes. *Nat Methods*. 9:103-109.

Huang, J., W. Zhou, W. Dong, A.M. Watson, and Y. Hong. 2009. Directed, efficient, and versatile modifications of the *Drosophila* genome by genomic engineering. *Proc Natl Acad Sci*. 106:8284-8289.

Kim, S., I. Gailite, B. Moussian, S. Luschnig, M. Goette, K. Fricke, M. Honemann-Capito, H. Grubmuller, and A. Wodarz. 2009. Kinase-activity-independent functions of atypical protein kinase C in Drosophila. *J Cell Sci*. 122:3759-3771.

Klein, T., and A.M. Arias. 1998. Interactions among Delta, Serrate and Fringe modulate Notch activity during Drosophila wing development. *Development*. 125:2951-2962.

Klose, S., D. Flores-Benitez, F. Riedel, and E. Knust. 2013. Fosmid-based structure-function analysis reveals functionally distinct domains in the cytoplasmic domain of *Drosophila* Crumbs. *G3 (Bethesda)*. 3:153-165.

Kumichel, A., K. Kapp, and E. Knust. 2015. A Conserved Di-Basic Motif of *Drosophila* Crumbs Contributes to Efficient ER Export. *Traffic*. 16:604-616.

Levy, P., and C. Larsen. 2013. Odd-skipped labels a group of distinct neurons associated with the mushroom body and optic lobe in the adult Drosophila brain. *J Comp Neurol*. 521:3716-3740.

Mettler, U., G. Vogler, and J. Urban. 2006. Timing of identity: spatiotemporal regulation of hunchback in neuroblast lineages of Drosophila by Seven-up and Prospero. *Development*. 133:429-437.

Richard, M., F. Grawe, and E. Knust. 2006. *D*PATJ plays a role in retinal morphogenesis and protects against light-dependent degeneration of photoreceptor cells in the *Drosophila* eye. *Dev Dyn*. 235:895-907.

Skeath, J.B., and S.B. Carroll. 1992. Regulation of proneural gene expression and cell fate during neuroblast segregation in the Drosophila embryo. *Development*. 114:939-946.

Wodarz, A., U. Hinz, M. Engelbert, and E. Knust. 1995. Expression of Crumbs confers apical character on plasma membrane domains of ectodermal epithelia of *Drosophila*. *Cell*. 82:67-76.

Wodarz, A., A. Ramrath, U. Kuchinke, and E. Knust. 1999. Bazooka provides an apical cue for Inscuteable localization in *Drosophila* neuroblasts. *Nature*. 402:544-547.


Biology Open • Supplementary information
